# Plasma-based longitudinal mutation monitoring as a potential predictor of disease progression in subjects with adenocarcinoma in advanced non-small cell lung cancer

**DOI:** 10.1186/s12885-020-07340-z

**Published:** 2020-09-15

**Authors:** John Jiang, Hans-Peter Adams, Maria Lange, Sandra Siemann, Mirjam Feldkamp, Sylvie McNamara, Sebastian Froehler, Stephanie J. Yaung, Lijing Yao, Aarthi Balasubramanyam, Nalin Tikoo, Christine Ju, H. Jost Achenbach, Rainer Krügel, John F. Palma

**Affiliations:** 1 Roche Sequencing Solutions, 4300 Hacienda Dr, Pleasanton & Potsdam, California, 94588 USA; 2 Signature Diagnostics GmbH, Hermannswerder 20A, 14473 Potsdam, Germany; 3 Roche Molecular Systems, 4300 Hacienda Dr., Pleasanton, CA 94588 USA; 4 Lungenklinik Lostau, Lindenstraße 2, 39291 Lostau, Germany; 5 Johanniter Krankenhaus im Fläming, Johanniterstraße 1, 14929 Treuenbrietzen, Germany

**Keywords:** Next-generation sequencing (NGS), Cell-free DNA (cfDNA), Circulating tumor DNA (ctDNA), Liquid biopsy, Non-small cell lung cancer (NSCLC)

## Abstract

**Background:**

Identifying and tracking somatic mutations in cell-free DNA (cfDNA) by next-generation sequencing (NGS) has the potential to transform the clinical management of subjects with advanced non-small cell lung cancer (NSCLC).

**Methods:**

Baseline tumor tissue (*n* = 47) and longitudinal plasma (*n* = 445) were collected from 71 NSCLC subjects treated with chemotherapy. cfDNA was enriched using a targeted-capture NGS kit containing 197 genes. Clinical responses to treatment were determined using RECIST v1.1 and correlations between changes in plasma somatic variant allele frequencies and disease progression were assessed.

**Results:**

Somatic variants were detected in 89.4% (42/47) of tissue and 91.5% (407/445) of plasma samples. The most commonly mutated genes in tissue were *TP53* (42.6%), *KRAS* (25.5%), and *KEAP1* (19.1%). In some subjects, the allele frequencies of mutations detected in plasma increased 3–5 months prior to disease progression. In other cases, the allele frequencies of detected mutations declined or decreased to undetectable levels, indicating clinical response. Subjects with circulating tumor DNA (ctDNA) levels above background had significantly shorter progression-free survival (median: 5.6 vs 8.9 months, respectively; log-rank *p* = 0.0183).

**Conclusion:**

Longitudinal monitoring of mutational changes in plasma has the potential to predict disease progression early. The presence of ctDNA mutations during first-line treatment is a risk factor for earlier disease progression in advanced NSCLC.

## Background

Lung cancer is the leading cause of cancer death [1]. Non-small cell lung cancer (NSCLC) is the most common type of lung cancer, comprising 80–85% of all lung cancers [2]. About 40% of lung cancers were diagnosed at advanced stage [3]. The 5-year relative survival rate of stage IV NSCLC is only 6% [2]. Over the past 20 years, treatments for advanced stage NSCLC have evolved from empirical cytotoxic therapies to more precision treatments including targeted therapies and immunotherapies. Therapies targeting specific genomic alterations, such as epidermal growth factor receptor (EGFR) mutations, anaplastic lymphoma kinase (ALK), ROS proto-oncogene 1, receptor tyrosine kinase (ROS1) fusions, have greatly improved NSCLC management [4]. Assessing therapy response and monitoring disease progression are critical components of providing the right therapy at the right time and further improving overall survival.

Liquid biopsies have emerged as a crucial tool in cancer management. When tumor cells undergo apoptosis or necrosis, DNA is shed into the bloodstream [5, 6]. This shed DNA (circulating tumor DNA [ctDNA]) contains gene mutations, representative of those found in primary and metastatic tumors [7–9]. The specific molecular changes identified in ctDNA could have diagnostic value and be used to predict therapy response and patient survival [10, 11]. The ctDNA can be harvested via blood collection, and due to the relative ease with which “liquid biopsies” are performed, it is possible to obtain serial samples and to analyze changes in tumor composition, and to monitor treatment response and disease progression over time [12, 13]. Moreover, because both primary and metastatic tumors shed DNA into the circulation, liquid biopsies are likely to provide a more comprehensive picture of the total cancer genome than traditional biopsies. As a consequence, the ability of ctDNA to assist in the diagnosis, treatment, and surveillance/monitoring of cancer has become an area of active research [14, 15].

Theoretically, liquid biopsies coupled with next-generation sequencing (NGS) can be used to monitor the evolution of NSCLC over time. If known resistance mutations emerge, the appropriateness of a prescribed treatment regimen could be reassessed [16]. The present analysis examined the ability of NGS coupled with ctDNA from serially acquired plasma samples to assess treatment response and/or disease progression and to explore the potential of ctDNA as a prognostic factor in subjects receiving first-line treatment for advanced NSCLC.

## Methods

### Study design and subjects

This was a retrospective sub-study of adults aged ≥18 years participating in the prospective German Time Series of Biomarkers in Lung Cancer (ZRLK) study. Study participants had not previously been treated systemically for advanced NSCLC, which was diagnosed per the Prevention, Diagnosis, Therapy, and Follow-up of Lung Cancer Interdisciplinary Guidelines from the German Respiratory Society and the German Cancer Society [17]. To be eligible for the sub-study, subjects had to have had histologically proven adenocarcinoma of the lung and at least 2 blood draws during first-line treatment (i.e., prior to clinical disease progression). All subjects provided written informed consent within the context of a prospective observational study, and the study adhered to Good Clinical Practice guidelines and Declaration of Helsinki principles.

Per study protocol, blood samples were collected at baseline, prior to each chemotherapy cycle, and about every 3 months during treatment-free intervals. Twenty mL of peripheral blood was collected in EDTA-coated tubes. Plasma was separated by centrifugation at 1500 x g for 15 min within 2 h of sample collection and stored at − 80 °C until DNA extraction. In total, 445 blood samples from the 71 patient cohort were collected, with minimum 2 and maximum 18 samples per patient. The median number of blood samples per patient was 6. Out of 71 subjects, 47 (66.2%) had a pre-treatment tissue sample available and 42 of the 47 (89.4%) had somatic variants detected. Distribution of baseline characteristics were compared between subjects with tissue biopsies (*n* = 47) and the remaining subjects (*n* = 24) by Wilcoxon rank-sum test, Pearson’s chi-square test, and Fisher’s exact test as appropriate.

Cell-free DNA (cfDNA) was extracted from 4 mL of plasma, and tumor DNA was isolated from formalin-fixed paraffin-embedded (FFPE) tumor tissue sections using the AVENIO cfDNA isolation kit (Roche, Branchburg, New Jersey) and KAPA Express Extract kit (Roche, Cape Town, South Africa), respectively. DNA yields were quantified by a Qubit fluorometer (ThermoFisher Scientific, Waltham, Massachusetts). The cfDNA yield had a range of 13 to 1164 ng with the median of 58 ng. The amount of isolated tumor tissue DNA was from 63 ng to 15,420 ng with a median of 1180 ng. cfDNA was sequenced using the AVENIO ctDNA Surveillance assay (Research Use Only; Roche, Branchburg, New Jersey), and tumor tissue DNA was sequenced using a FFPET assay (AVENIO Tumor Tissue Surveillance Kit, Research Use Only; in development; Roche, Branchburg, New Jersey). The median input mass for FFPE samples was 50 ng (range 9–200 ng) and median input mass for plasma samples was 20 ng (range 10–50 ng). High-throughput sequencing was performed on the Illumina NextSeq 500 instrument (Illumina, San Diego, California). Both the AVENIO ctDNA Surveillance Kit and the prototype of AVENIO tumor tissue assays, which are based on Cancer Personalized Profiling by Deep Sequencing (CAPP-Seq) technology [15], utilize molecular barcoding and a hybrid-capture methodology to interrogate selected regions and recurrent mutations from 197 genes commonly mutated in cancer, including 17 important cancer-related genes (e.g., *EGFR*, *KRAS*, *BRAF*, *TP53*). The average de-duplicated sequencing depth of ctDNA and FFPE tumor tissue DNA was 2779 and 1116, respectively. Somatic variants were identified using the AVENIO ctDNA Analysis Software (Roche, Branchburg, New Jersey), which incorporates integrated digital error suppression [18] to remove polymerase chain reaction duplicates and stereotypical errors.

### Identification of variants in plasma-derived ctDNA and FFPE tumor tissue DNA

Somatic variants in cfDNA were derived by filtering out predicted germline variants using an algorithm that is able to detect putative somatic variants with allele frequencies < 30% (not excluding whitelist mutation); this is possible because the algorithm relies on relevant information from multiple somatic mutations rather than on an allele frequency cut-off (described below). After using molecular barcoding and background polishing algorithms (adapted from those described in Newman et al., 2016) [19], the AVENIO ctDNA Surveillance Kit adaptively sets the lower threshold for variant calling based both on the background of a sample and error profile at a given position; no global lower threshold for allele frequencies is applied. Somatic variants in FFPE tumor tissue DNA were derived by post-call filters according to the following criteria: (1) variants had to be in non-repetitive regions of the genome, (2) variants are not predicted to be germline variants (described below), (3) the population frequencies of variants had to be < 1% in the Exome Aggregation Consortium (ExAC) database, and (4) the allele frequencies of variants had to be > 5% (or > 3% if variants were well known hot-spot mutations). Both synonymous and non-synonymous somatic variants were selected for further analysis.

### Germline-variant filtering for each subject

To accurately identify somatic mutations in the absence of matched normal sequences, a machine-learning algorithm (CSMutan) was developed that leveraged mutation databases (ExAC, Single Nucleotide Polymorphism database, 1000 Genomes Project, Catalogue of Somatic Mutations in Cancer, Cancer Genome Atlas) and variant allele frequencies. Using in-house sequencing data of plasma samples whose germline single nucleotide polymorphisms have been identified by comparison with matched normal DNA, we built a classifier to distinguish germline variants from somatic variants with a set of features derived from databases and samples’ variants allele frequency. The detail description of the machine learning algorithm could be found in filed patent application [20]. This classifier was then used to predict germline variant profiles for individual subjects.

### ctDNA detection method

The presence (ctDNA – positive) vs absence (ctDNA-negative) of mutant ctDNA was evaluated using a previously described Monte Carlo method [15] indicating whether a given set of variants for each sample was significantly higher than background across the sequenced genomic regions. For a given plasma sample, we started with a set of variants (n), which were high-confidence somatic mutations identified in the matched baseline tissue sample. Using Monte Carlo simulation, we compared the mean allele frequency of the reports against the null distribution of background allele alterations, which most are background sequencing errors, in the plasma sample. In the simulation, we conducted 10,000 iterations, each of which randomly picked n background allele alterations and calculated their mean allele frequency. The empirical *p*-value of the plasma sample was determined as percentile of mean allele frequency regarding to the null distribution of the background allele alterations. Plasma samples, whose variants with empirical *p*-values from the Monte Carlo method were less than 0.01, were considered to be ctDNA-positive and otherwise ctDNA-negative.

### Correlation of allele frequencies to clinical response

Subjects underwent routine CT imaging at baseline and after every two therapy cycles (not all data available); if clinically indicated, the frequency of CT imaging was adjusted. CT scans were evaluated by a reference radiologist at the Thoraxklinik, Ruprecht-Karls-University at Heidelberg according to Response Evaluation Criteria in Solid Tumors (RECIST) v1.1 [21]. The relationship between total ctDNA level or the presence/absence of ctDNA mutations and radiological response to treatment was assessed. Total ctDNA level was quantified by summing the allele frequency of each variant detected and normalizing to the total amount of input cfDNA. Progression-free survival (PFS) time was computed as difference in days between date of histological diagnosis and date of diagnosis of a progression of disease event defined by RECIST criteria or date of death of any course. The PFS time was censored for all other subjects at the date of the last imaging assessment. PFS was analyzed descriptively using the Kaplan–Meier method. Individual Cox proportional hazards models were used to assess the significance of each potential adjustment factor (age, sex, ECOG (0 vs. 1 or 2), smoking status (current smoker vs. never smoker vs. quit smoking), and disease stage (IIIA vs. IIIB vs. IV) with PFS. None of these factors were significantly associated with PFS. Two final unadjusted Cox proportional hazard regression models were used to calculate hazard ratios (HRs) with 95% confidence intervals (CIs) for ctDNA mutation status for the first available post-treatment plasma sample and the last available post-treatment plasma sample. Molecular progression was defined as mutational allele frequencies with consecutive increases from baseline.

## Results

In total, 71 subjects with NSCLC eligible for first-line treatment were enrolled between February 2014 and June 2016. The baseline characteristics of these subjects are summarized in Table 1. The average age was 62.5 years, and the majority of subjects were male (63.4%) and had adenocarcinoma (96%). In total, 5.6, 11.3, and 83.1% of subjects presented with stage IIIA, IIIB, and IV disease, respectively. Cisplatin/carboplatin plus pemetrexed were the chemotherapy regimen for the majority of subjects. Some patients also received Bevacizumab in addition to chemo (Supplementary Table 1). From these 71 subjects, 445 longitudinal plasma samples (range of 2–18 samples per subject) were collected over an average of 5 months (range, 1–25). Forty-seven of the 71 subjects had baseline tissue sample available. Baseline characteristics were not significantly different between subjects with tissue biopsies (*n* = 47) compared to the remaining subjects (*n* = 24), suggesting that the 47 subjects did not differ from the total study cohort. (Table 1).

**Table 1:** Baseline Demographics and Disease Characteristics

	Total Study Cohort(***n*** = 71)	Subset of Subjects with Pretreatment Tissue Biopsies(n = 47)	Remaining Subjects(n = 24)	***P***-value
Age (years)				0.9466
Mean (SD)	62.54 (10.0)	62.43 (9.2)	62.75 (11.6)	
Median	62	62	61.5	
Age, n(%)				0.8108
< 70	52 (73)	34 (72)	18 (75)	
> = 70	19 (27)	13 (28)	6 (25)	
Sex, n(%)				0.5282
Female	26 (37)	16 (34)	10 (42)	
Male	45 (63)	31 (66)	14 (58)	
ECOG Status, n(%)				0.1672
0	22 (31)	17 (36)	5 (21)	
1	44 (62)	28 (60)	16 (67)	
2	3 (4)	2 (4)	1 (4)	
3	2 (3)	–	2 (8)	
Stage, n(%)				0.3136
IIIA	4 (6)	2 (4)	2 (8)	
IIIB	8 (11)	7 (15)	1 (4)	
IV	59 (83)	38 (81)	21 (88)	
Histology, n(%)				0.4137
Adenocarcinoma	68 (96)	45 (96)	23 (96)	
Adenocarcinoma|Large cell carcinoma^a^	1 (1)	–	1 (4)	
Adenocarcinoma|Squamous cell carcinoma^a^	2 (3)	2 (4)	–	
1 L Therapy Received, n(%)				0.8582
Chemotherapy Only^b^	31 (44)	21 (45)	10 (42)	
Chemo+Radiation^c^	25 (35)	17 (36)	8 (33)	
Chemo+Targeted^d^	9 (13)	6 (13)	3 (13)	
Chemo+Radiation+Targeted^d^	6 (8)	3 (6)	3 (13)	
Smoking Status, n(%)				0.8495
Never Smoker	11 (15)	6 (13)	5 (21)	
Quit more than 5 years ago	9 (13)	6 (13)	3 (13)	
Quit less than 5 years ago	9 (13)	6 (13)	3 (13)	
Current Smoker	42 (59)	29 (62)	13 (54)	

ECOG, Eastern Cooperative Oncology Group; SD, standard deviation. ^a^All subjects received an initial diagnosis of adenocarcinoma; however, additional histology was reported after re-biopsy; ^b^Cisplatin/carboplatin plus pemetrexed; ^c^Radiation included for brain or bone metastatic sites; ^d^Targeted therapy was bevacizumab in 13 subjects, erlotinib plus chemotherapy and radiation in 1 subject, and 1 subject received Nivolumab after chemotherapy and bevacizumab

Somatic variations were detected in 91.5% (407/445) of plasma samples, with an average of five mutations per sample. Of the 47 subjects with available tissue biopsies, somatic mutations were detected in 42 (89.4%), with *TP53*, *KRAS*, and *KEAP1* being the most frequently mutated genes (Table 2). The average number of mutations detected in tissue samples was six. The mean allele frequency for mutations detected in plasma-derived ctDNA was 1.74%, ranging from 0.03 to 45.28%; the corresponding value in FFPE tumor tissue DNA was 21.7%, ranging from 3.29 to 86.82%. The most frequent mutations in all ctDNA samples were in *TP53* (27.9%), *KRAS* (21.3%), and *NPAP1* (16.4%) (Supplementary Table 2). In baseline ctDNA samples, the top mutated genes were *TP53* (43.1%), *KRAS* (29.4%), *NPAP1* (29.4%), *LRRTM4* (17.6%), *CSMD3* (15.7%), and *MET* (15.7%) (Supplementary Table 3). On average75.2% of somatic variants identified in tissue were also identified in the matched baseline ctDNA plasma sample.

**Table 2:** Gene Mutation Frequencies in Pre-treatment Tissue Biopsies

Gene	Subset of Subjects with Pre-Treatment Tissue Biopsies (N = 47)
*TP53*	20 (42.6)
*KRAS*	12 (25.5)
*KEAP1*	9 (19.1)
*BRAF*	6 (12.8)
*STK11*	5 (10.6)
*ERBB2*	5 (10.6)
*EGFR*	2 (4.2)

Data are n (%)

**Fig. 1:**
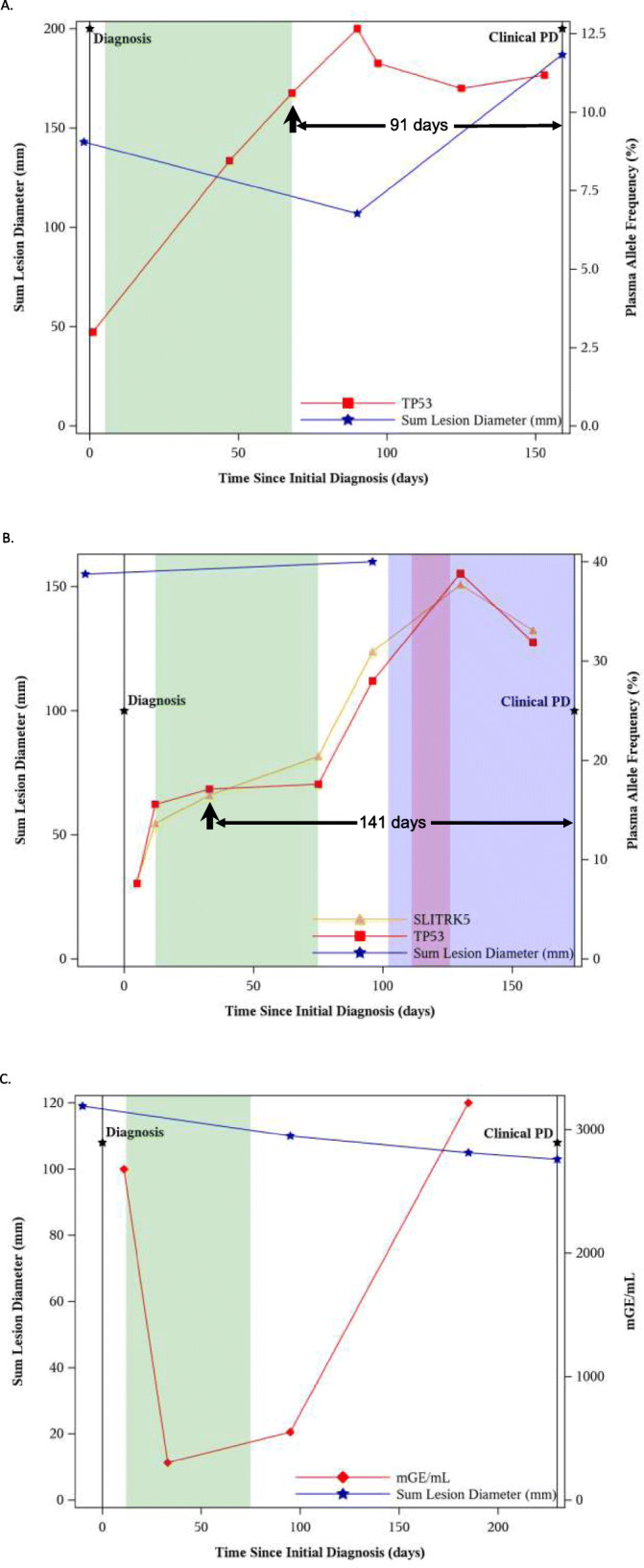
Correlation between allele frequencies in ctDNA and clinical disease in a subject with **a** a single somatic mutation and **b** two somatic mutations. **c** Correlation between total ctDNA load and clinical disease. The duration of treatment with chemotherapy is represented by the green band, radiation by the beige band, and targeted therapy by the light purple band. The overlap in radiation and targeted therapy is represented by the dark purple band. ctDNA, circulating tumor DNA; mGE, mutated genome equivalent; PD, progressive disease

By utilizing both the AVENIO ctDNA Surveillance Kit and the tumor tissue Surveillance kit, we were able to track tumor mutation changes over time. In the two patient examples forthwith with corresponding imaging data, detection of ctDNA preceded radiographic progression (per RECIST 1.1). In some subjects, the allele frequencies of mutations detected in ctDNA increased consecutively 3–5 months before clinical evidence of disease progression. For example, in one subject who had been treated with carboplatin plus pemetrexed, the allele frequency of mutations in *TP53* (CDS mutation: c.712 T > G) increased from 3.0% at diagnosis to 8.5% at day 47 and to 10.6% at day 68 post-treatment. However, the CT scans did not show evidence of disease progression (75% increase in the diameters of target lesions plus new lesion development in the liver) until day 159—a difference of 91 days (Fig. 1a). Similar trends were uncovered in another subject in whom mutations in *TP53* and *SLITRK5* were detected. Molecular progression (defined as a consecutive increase in mutation allele frequencies) was apparent on day 33 post-diagnosis of metastatic disease, but clinical disease progression was not seen until day 174 (Fig. 1b). We also examined the relationship between total ctDNA level, which sum up all mutant molecules detected in plasma sample, and treatment response. For example, the analysis had shown that the ctDNA level from one study subject changed with therapy administration and clinical disease progression (specifically, development of a new non-target lesion) (Fig. 1c).

**Fig. 2:**
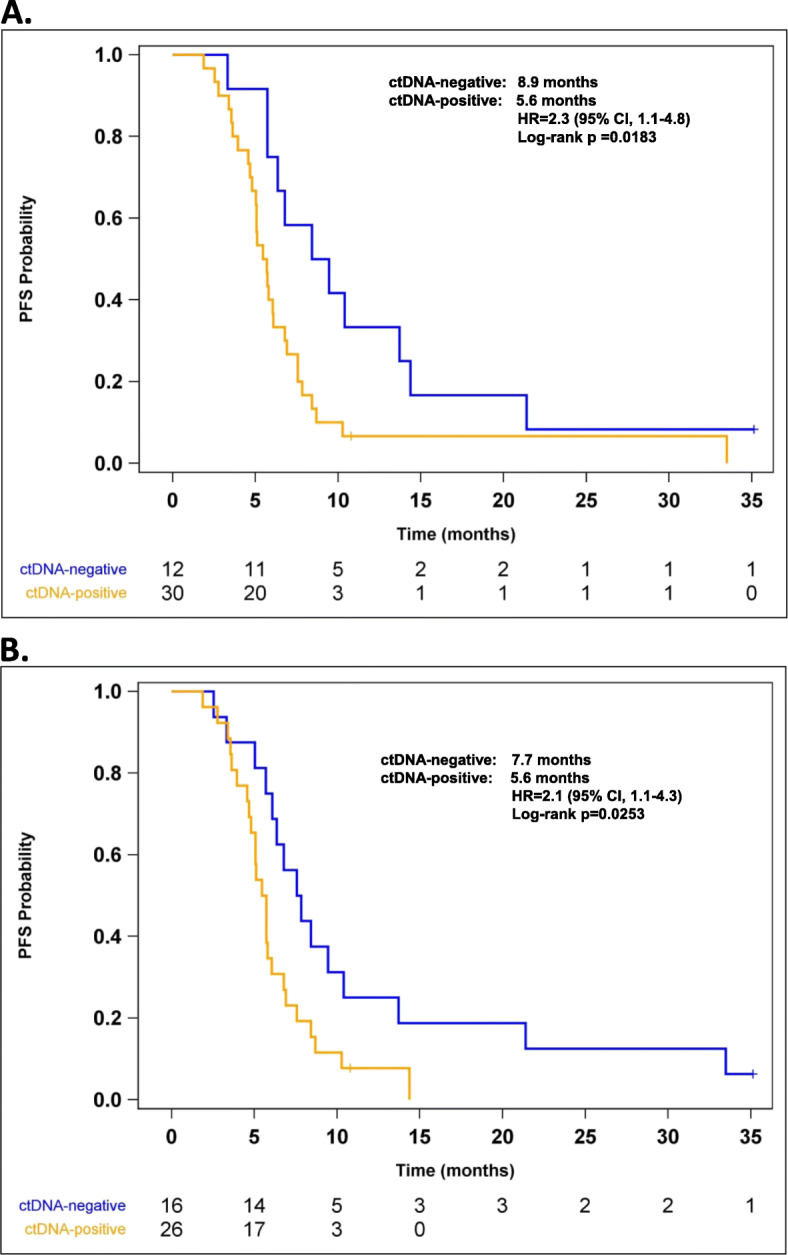
Median PFS in subjects stratified by the presence/absence of mutations in ctDNA isolated from the **a** first available post-treatment plasma sample and **b** last available post-treatment plasma sample. CI, confidence interval; ctDNA, circulating tumor DNA; HR, hazard ratio; PFS, progression-free survival

The prognostic value of cancer mutations in plasma-derived ctDNA was further evaluated. When stratifying subjects by ctDNA mutation status in the first available post-treatment plasma sample, we found that ctDNA-positive subjects vs. ctDNA-negative subjects were associated with reduced PFS. Median PFS was 5.6 months in the ctDNA-positive group and 8.9 months in the ctDNA-negative group. The HR for the comparison of the two groups (ctDNA-positive vs. ctDNA-negative) was 2.3 (95% CI, 1.1–4.8; log-rank *p* = 0.0183) (Fig. 2a). A sensitivity analysis in which ctDNA mutation status was determined using the last available plasma sample yielded similar results (Fig. 2b). Analysis using individual Cox proportional hazards models had demonstrated that age, gender, ECOG status, smoking status, and disease stage were not significantly associated with disease progression, as shown in Table 3.

**Table 3:** Association of Potential Adjustment Factors with Progression Free Survival

		Unadjusted	
**Variable**	**Level**	**HR (95% CI)**	**P**
Age		0.99 (0.96, 1.03)	0.6628
Sex	Female (Reference = Male)	1.24 (0.63, 2.46)	0.5350
ECOG	0 (Reference = 1 or 2)	1.22 (0.63, 2.37)	0.5554
Smoking Status	Current Smoker (Reference = Quit Smoking)	0.98 (0.48, 2.00)	0.2816
	Never Smoker (Reference = Quit Smoking)	0.37 (0.10, 1.37)	
Disease Stage	IIIA (Reference = IV)	1.07 (0.25, 4.54)	0.7842
	IIIB (Reference = IV)	1.38 (0.56, 3.37)	

Individual unadjusted Cox proportional hazards models were used for each potential adjustment factor

Global *p*-values presented for categorical variables with more than 2 levels

## Discussion

In this study, longitudinal monitoring analysis of somatic mutations in plasma samples from subjects receiving first-line treatment for advanced adenocarcinoma of the lung was explored. The average number of mutations detected in tissue and plasma was very similar, and about 90% of all tumor tissue and plasma sample tested had 1 or more variants identified. In the cohort of this study subjects, the most frequently detected mutations in FFPE tumor tissue DNA were *TP53*, *KRAS*, and *KEAP1*, which is consistent with the findings of Chaudhuri et al. [22] The prevalence of EGFR mutation in this study (4.2%) was lower than that of TCGA data [18]. This may be due to the small tissue sample size and the nature of this prospective collection that part of the EGFR mutated population were enrolled in other clinical studies and tissue samples were not available for this analysis.

Serial plasma sampling provides an opportunity to detect mutations emerging over time and to monitor disease progression based on the presence or change of mutant molecules. Moreover, liquid biopsies contain the genomes of primary and metastatic tumors [14]. This contrasts with traditional biopsies, which excise tissue from only a single tumor location and which are thus subject to tumor and genomic heterogeneity. Although ctDNA levels in blood can be low, the sequencing of multiple plasma samples—each of which potentially contains the genomes of multiple metastatic tumors— increases the likelihood of detecting somatic, disease-associated mutations, including low-frequency variants.

Through use of the AVENIO ctDNA Surveillance Kit (Research Use Only), which enabled us to assess changes in the mutation status of both single and multiple cancer genes over time, we found the presence of ctDNA in post-treatment plasma samples to be a risk factor for disease progression, independent of clinical parameters. Moreover, molecular relapse in 2 patient cases was shown to precede clinical relapse by 3–5 months, a finding supported by the work of others [15, 22–24]. For example, Phallen J *et al* [23] have demonstrated that ctDNA non-responders had a significantly shorter median progression-free survival compared to ctDNA responders in the metastatic NSCLC population treated with targeted tyrosine kinase inhibitors. A recent study in KRAS mutated NSCLC patients had also shown that KRAS mutation presence or dynamic change in post treatment plasma was significantly associated with increased probability of experiencing progressive disease, and PFS and OS [25]. Therefore, changing therapy when ctDNA level is increasing, before radiographic progression, might potentially improve the clinical outcome. Clinical studies are needed to explore this potential.

We also found total ctDNA level to be prognostic of disease progression and ctDNA-positivity vs ctDNA-negativity to be associated with reduced median PFS. Collectively, these preliminary results suggest that deep sequencing of serially acquired liquid biopsies from subjects with advanced NSCLC has the potential to facilitate longitudinal disease monitoring and to predict disease progression earlier than radiologic scans. The identification of resistance clones or signatures prior to clinical disease progression may provide an opportunity to transition sooner to alternative therapy, but additional studies are needed to validate this concept, and prospective clinical trials are necessary to demonstrate its clinical utility.

## Conclusions

The presence of ctDNA during treatment of late stage NSCLC is a significant risk factor for survival. Total ctDNA level may reflect disease burden at a specific time point. ctDNA increase could be detected months before clinical progression, and this information could potentially be used for disease monitoring in conjunction with imaging which might have a positive impact on patient outcome. ctDNA assessment may provide significant value to improve therapy selection, disease surveillance and monitoring, and drug resistant mutation detection.

## Supplementary information

**Additional file 1: Supplementary Table 1.** Treatment regimens of study subjects received.

**Additional file 2: Supplementary Table 2.** Somatic variants detected in all plasma samples.

**Additional file 3: Supplementary Table 3.** Somatic variants detected in baseline plasma samples.

## Data Availability

The datasets generated and/or analysed during the current study are not publicly available due to General Data Protection Regulation and sensitive genetic information.

## Abbreviations

CAPP-Seq: Cancer Personalized Profiling by Deep Sequencing
cfDNA: Cell-free DNA
CI: Confidence interval
ctDNA: circulating tumor DNA
ExAC: Exome Aggregation Consortium
FFPE: Formalin-fixed paraffin-embedded
HR: Hazard ratio
NGS: Next-generation sequencing
NSCLC: Non-small cell lung cancer
PFS: Progression-free survival
RECIST: Response Evaluation Criteria in Solid Tumors
ZRLK: German Time Series of Biomarkers in Lung Cancer
