# Supplementary material for: Plasma-based longitudinal mutation monitoring as a potential predictor of disease progression in subjects with adenocarcinoma in advanced non-small cell lung cancer
Source: BMC Cancer. 2020 Sep 15;20:885. doi: 10.1186/s12885-020-07340-z (PMC7493404; doi:10.1186/s12885-020-07340-z)
Supplement: Supplementary file 1 — Additional file 1: Supplementary Table 1. Treatment regimens of study subjects received. [file 12885_2020_7340_MOESM1_ESM.docx]

Supplementary Table 1. Treatment regimens of study subjects received
