# Supplementary material for: Plasma-based longitudinal mutation monitoring as a potential predictor of disease progression in subjects with adenocarcinoma in advanced non-small cell lung cancer
Source: BMC Cancer. 2020 Sep 15;20:885. doi: 10.1186/s12885-020-07340-z (PMC7493404; doi:10.1186/s12885-020-07340-z)
Supplement: Supplementary file 3 — Additional file 3: Supplementary Table 3. Somatic variants detected in baseline plasma samples. [file 12885_2020_7340_MOESM3_ESM.docx]

Supplementary Table 3. Somatic variants detected in baseline plasma samples

| **Gene** | **Frequency** | **Count** |
| --- | --- | --- |
| TP53 | 0.43137255 | 22 |
| KRAS | 0.29411765 | 15 |
| NPAP1 | 0.29411765 | 15 |
| LRRTM4 | 0.17647059 | 9 |
| CSMD3 | 0.15686275 | 8 |
| MET | 0.15686275 | 8 |
| BRCA1 | 0.1372549 | 7 |
| BRCA2 | 0.1372549 | 7 |
| RET | 0.1372549 | 7 |
| APC | 0.11764706 | 6 |
| EGFR | 0.11764706 | 6 |
| KEAP1 | 0.11764706 | 6 |
| LRRC7 | 0.11764706 | 6 |
| ZFPM2 | 0.11764706 | 6 |
| BCHE | 0.09803922 | 5 |
| BRAF | 0.09803922 | 5 |
| CHRM2 | 0.09803922 | 5 |
| DCAF12L1 | 0.09803922 | 5 |
| ERBB2 | 0.09803922 | 5 |
| FAM135B | 0.09803922 | 5 |
| FBXL7 | 0.09803922 | 5 |
| HTR1E | 0.09803922 | 5 |
| LPPR4 | 0.09803922 | 5 |
| NAV3 | 0.09803922 | 5 |
| SLITRK4 | 0.09803922 | 5 |
| SLITRK5 | 0.09803922 | 5 |
| USP29 | 0.09803922 | 5 |
| ZNF521 | 0.09803922 | 5 |
| DCAF4L2 | 0.07843137 | 4 |
| KIT | 0.07843137 | 4 |
| LRRTM1 | 0.07843137 | 4 |
| P2RY10 | 0.07843137 | 4 |
| PDZRN3 | 0.07843137 | 4 |
| POM121L12 | 0.07843137 | 4 |
| SLC8A1 | 0.07843137 | 4 |
| SLITRK1 | 0.07843137 | 4 |
| TRIM58 | 0.07843137 | 4 |
| ALK | 0.05882353 | 3 |
| DCAF12L2 | 0.05882353 | 3 |
| EPHA5 | 0.05882353 | 3 |
| GPR139 | 0.05882353 | 3 |
| HCN1 | 0.05882353 | 3 |
| KIAA1211 | 0.05882353 | 3 |
| LRFN5 | 0.05882353 | 3 |
| MKRN3 | 0.05882353 | 3 |
| NLRP3 | 0.05882353 | 3 |
| PDGFRA | 0.05882353 | 3 |
| PGK2 | 0.05882353 | 3 |
| ROS1 | 0.05882353 | 3 |
| STK11 | 0.05882353 | 3 |
| TMEM200A | 0.05882353 | 3 |
| TRPS1 | 0.05882353 | 3 |
| ADAMTS16 | 0.03921569 | 2 |
| ASTN1 | 0.03921569 | 2 |
| C6orf118 | 0.03921569 | 2 |
| CDH9 | 0.03921569 | 2 |
| CPXCR1 | 0.03921569 | 2 |
| DCLK1 | 0.03921569 | 2 |
| EYS | 0.03921569 | 2 |
| FAM71B | 0.03921569 | 2 |
| GRIK3 | 0.03921569 | 2 |
| GRM1 | 0.03921569 | 2 |
| HTR2C | 0.03921569 | 2 |
| KCNB2 | 0.03921569 | 2 |
| KPRP | 0.03921569 | 2 |
| LRP1B | 0.03921569 | 2 |
| NMUR1 | 0.03921569 | 2 |
| PCDH15 | 0.03921569 | 2 |
| PKHD1L1 | 0.03921569 | 2 |
| THSD7A | 0.03921569 | 2 |
| ZIC1 | 0.03921569 | 2 |
| ZIC4 | 0.03921569 | 2 |
| ASTN2 | 0.01960784 | 1 |
| AVPR1A | 0.01960784 | 1 |
| C6 | 0.01960784 | 1 |
| CACNA1E | 0.01960784 | 1 |
| CDH12 | 0.01960784 | 1 |
| CDH18 | 0.01960784 | 1 |
| CDH8 | 0.01960784 | 1 |
| CDKN2A | 0.01960784 | 1 |
| CNTN5 | 0.01960784 | 1 |
| CNTNAP2 | 0.01960784 | 1 |
| CSMD1 | 0.01960784 | 1 |
| CTNNB1 | 0.01960784 | 1 |
| DCSTAMP | 0.01960784 | 1 |
| DDI1 | 0.01960784 | 1 |
| DOCK3 | 0.01960784 | 1 |
| DSC3 | 0.01960784 | 1 |
| EPHA6 | 0.01960784 | 1 |
| FAT1 | 0.01960784 | 1 |
| FCRL5 | 0.01960784 | 1 |
| FOXG1 | 0.01960784 | 1 |
| GJA8 | 0.01960784 | 1 |
| GRIN3B | 0.01960784 | 1 |
| GRM5 | 0.01960784 | 1 |
| GRM8 | 0.01960784 | 1 |
| HCRTR2 | 0.01960784 | 1 |
| HS3ST4 | 0.01960784 | 1 |
| HS3ST5 | 0.01960784 | 1 |
| HTR1A | 0.01960784 | 1 |
| IFI16 | 0.01960784 | 1 |
| KCNA5 | 0.01960784 | 1 |
| KCNJ3 | 0.01960784 | 1 |
| MMP16 | 0.01960784 | 1 |
| NEUROD4 | 0.01960784 | 1 |
| NR0B1 | 0.01960784 | 1 |
| NRAS | 0.01960784 | 1 |
| NRXN1 | 0.01960784 | 1 |
| PDYN | 0.01960784 | 1 |
| PIK3CG | 0.01960784 | 1 |
| RFX5 | 0.01960784 | 1 |
| SEMA5B | 0.01960784 | 1 |
| SLC18A3 | 0.01960784 | 1 |
| SOX9 | 0.01960784 | 1 |
| SPTA1 | 0.01960784 | 1 |
| TIAM1 | 0.01960784 | 1 |
| TNN | 0.01960784 | 1 |
| TNR | 0.01960784 | 1 |
| WBSCR17 | 0.01960784 | 1 |
